# Four new species of the genus *Otacilia* Thorell, 1897 from Hunan Province, China (Araneae, Phrurolithidae)

**DOI:** 10.3897/zookeys.620.7982

**Published:** 2016-09-29

**Authors:** Chi Jin, Lina Fu, Xiangchu Yin, Feng Zhang

**Affiliations:** 1 Key Laboratory of Invertebrate Systematics and Application, College of Life Sciences, Hebei University, Baoding, Hebei 071002, China; 2 Northwest Plateau Institute of Biology, Chinese Academy of Sciences, Xining, 810001, China

**Keywords:** Description, diagnosis, etymology, morphology, taxonomy

## Abstract

Four new species of the genus *Otacilia* Thorell, 1897 are reported from Hunan Province, China: *Otacilia hippocampa* **sp. n.**, *Otacilia yangmingensis* **sp. n.**, *Otacilia curvata* **sp. n.**, and *Otacilia submicrostoma* **sp. n.** All new species are described based on both sexes. In addition, the 55 known *Otacilia* species are divided into four species groups.

## Introduction

Phrurolithidae Banks, 1892 was elevated to family rank by Ramírez (2014), consistent with the suggestion of Deeleman-Reinhold (2001). The family is currently represented by 211 species belonging to 14 genera worldwide. Of these, four genera and 65 species are recorded from China (World Spider Catalog 2016, Fu et al. 2015, Fu et al. 2016a, b). The Phrurolithidae are mostly ground-dwelling spiders living in leaf litter, especially bamboo leaves, woody debris or on the forest floor, very few species are found in the canopy (Deeleman-Reinhold 2001; Fu et al. 2014).

*Otacilia* Thorell, 1897 is one of the species-rich genera of the family comprising 55 species distributed in south-east Asia and east Asia. Among these *Otacilia* species, 35 are reported from China (Fu et al. 2015, Fu et al. 2016a, Fu et al. 2016b). The genus *Otacilia* was established by Thorell (1897) with description of *Otacilia armatissima* based on a single female specimen from Myanmar.

The genus *Otacilia* is closely related to *Phrurolithus* C. L. Koch, 1839, comprising 74 species mostly distributed in the holarctic region. Until now, there is no clear way to differentiate between *Otacilia* and *Phrurolithus*. The diagnostic characters provided by Kamura (2005) were inaccurate (Wang et al. 2015), and the differences listed by Jäger and Wunderlich (2012) were also not distinct with the addition of more new species of these two genera.

Wang et al. (2015) listed ten Chinese *Otacilia* species in two groups. Subsequently, Fu et al. (2016b) reviewed the 31 Chinese *Otacilia* species and agreed with Wang et al.’s (2015) assignment and also established a third species group to accommodate the *Otacilia* species: the *armatissima* group, the *revoluta* group, and the *pseudostella* group.

While examining the collections from Hunan Province, China, some *Otacilia* specimens were found that differed from the currently known *Otacilia* species. They are identified as four new species, *Otacilia hippocampa* sp. n., *Otacilia yangmingensis* sp. n., *Otacilia curvata* sp. n., and *Otacilia submicrostoma* sp. n., and are described and illustrated here.

## Material and methods

The terminology used follows Jäger and Wunderlich (2012). All measurements given in the text are in millimeters. Total length is the sum of the carapace and abdomen lengths, regardless of the pedicel. Eye sizes were measured as the maximum diameter in dorsal or frontal view. Leg measurements are shown as: total length (femur, patella, tibia, metatarsus, tarsus). Epigyne were removed and cleared in a warm solution of 10% potassium hydroxide (KOH), transferred to ethanol and temporarily mounted for drawing. All specimens are preserved in 75% alcohol and were examined, drawn, and measured under a Leica M205A stereomicroscope equipped with an Abbe drawing device. Photographs were taken using a Leica M205A stereomicroscope equipped with a DFC450 CCD. The specimens are deposited in the Museum of Hebei University, Baoding, China (MHBU).

### Abbreviations

ALE
 anterior lateral eyes 


AME
 anterior median eyes 


a.s.l.
 above sea level 


B
 bursa 


C
 conductor 


CD
 copulatory duct 


CO
 copulatory opening 


CT
 connecting tube 


DTA
 dorsal tibial apophysis 


E
 embolus 


FA
 femoral apophysis 


FD
 fertilization duct 


GA
 glandular appendage 


MOA
 median ocular area 


MP
 median plate 


PLE
 posterior lateral eyes 


PME
 posterior median eyes 


RTA
 retrolateral tibial apophysis 


S
 spermatheca 


TA
 tegular apophysis 


## Taxonomy

### Phrurolithidae Banks, 1892

#### Otacilia

Taxon classificationAnimaliaAraneaePhrurolithidae

Thorell, 1897

##### Diagnosis.

Chelicerae each with two bristles (rarely with one bristle) on anterior side; leg formula: 4123 (rarely 1423); spination: femora I–II d 0–2, III–IV d 0–1, I pl 3–6, II pl 0–3; tibiae I–II usually with 6–8 pairs of ventral spines; tibia I always one more rv than pv spine and tibia II always one more pv than rv spine; metatarsi I–II usually with 3–4 pairs of ventral spines, and always one more pv than rv spine.

Male palp: femur with ventral apophysis or hump; tibia usually with single strong RTA, some species with BTA or DTA; embolus hook-shaped or needle-like, originating antero-prolaterally; tegular apophysis sclerotized or transparent, present or absent, antero-retrolaterally located; conductor membranous, well developed or absent. Female genitalia: epigynal median plate distinct or absent; vulva with pair of transparent bursae anteriorly and pair of strongly sclerotized spermathecae posteriorly.

##### Species groups of *Otacilia*.

After reviewing 59 *Otacilia* species (including the four new Chinese species described in this paper), the grouping was revised and the current species assigned to four groups based on assessment of Fu et al. (2016b). The *revoluta* group was divided into two new groups, the *longituba* group (16 species) and the *ambon* group (two species). The *armatissima* group (29 species) and the *pseudostella* group (nine species) were preserved and updated. Three species were not assigned to any group because of their poor original description and figures or peculiar structure: *Otacilia luzonica* (Simon, 1898) (female is unknown; description and figures are not diagnostic), *Otacilia papilla* Dankittipakul & Singtripop, 2014 (male is unknown; epigyne medially with lobe and absence of bursae) and *Otacilia paracymbium* Jäger & Wunderlich, 2012 (female is unknown; cymbium with paracymbium).

Here the male and female diagnostic characters are listed for each species group, followed by a list of all of the included *Otacilia* species (Table 1).

**Table 1.** Definition of species groups of *Otacilia* species, together with lists of included species (species marked with an asterisk are reported from China).

Species group name	Diagnostic Character	Included Species
*armatissima*	1) Palpal organ with a distinct sclerotized TA or membranous conductor; embolus hook-shaped. 2) Epigyne with a distinct median plate, without concavity; CO slocated anteriorly or medially, higher than the spermathecae; CD (the left one from the ventral view) anti-clockwise from the CO; connecting tube usually crescent-shaped; spermathecae separated by more than half a spermatheca’s diameter.	1) *Otacilia armatissima* Thorell, 1897 2) *Otacilia bawangling* Fu, Zhang & Zhu, 2010* 3) *Otacilia biarclata* Fu, He & Zhang, 2015* 4) *Otacilia bicolor* Jäger & Wunderlich, 2012 5) *Otacilia florifera* Fu, He & Zhang, 2015* 6) *Otacilia forcipata* Yang, Wang & Yang, 2013* 7) *Otacilia foveata* (Song, 1990)* 8) *Otacilia fujiana* Fu, Jin & Zhang, 2014* 9) *Otacilia hengshan* (Song, 1990)* 10) *Otacilia jianfengling* Fu, Zhang & Zhu, 2010* 11) *Otacilia kao* Jäger & Wunderlich, 2012 12) *Otacilia komurai* (Yaginuma, 1952)* 13) *Otacilia limushan* Fu, Zhang & Zhu, 2010* 14) *Otacilia liupan* Hu & Zhang, 2011* 15) *Otacilia luna* (Kamura, 1994) 16) *Otacilia lynx* (Kamura, 1994)* 17) *Otacilia macrospora* Fu. Zhang & Zhang, 2016* 18) *Otacilia obesa* Fu. Zhang & Zhang, 2016* 19) *Otacilia onoi* Deeleman-Reinhold, 2001 20) *Otacilia papilion* Fu, Zhang & Zhang, 2016* 21) *Otacilia pyriformis* Fu, Zhang & Zhang, 2016* 22) *Otacilia sinifera* Deeleman-Reinhold, 2001 23) *Otacilia songi* Wang et al., 2015* 24) *Otacilia subliupan* Wang et al., 2015* 25) *Otacilia taiwanica* (Hayashi & Yoshida, 1993)* 26) *Otacilia truncata* Dankittipakul & Singtripop, 2014 27) *Otacilia yangi* Zhang, Fu & Zhu, 2009* 28) *Otacilia hippocampa* sp. n.* 29) *Otacilia yangmingensis* sp. n.*
*ambon*	1) Palpal organ without a distinct TA; the bulb is not pyriform but oval; embolus claw-like. 2) Epigyne without median plate and concavity; Cos located posterior, lower than the spermathecae; spermathecae well separated from each other by more than three spermatheca’s diameter.	1) *Otacilia ambon* Deeleman-Reinhold, 2001 2) *Otacilia revoluta* (Yin et al., 2004)*
*longituba*	1) Palpal organ without a distinct TA; conductor well developed or degenerated; embolus needle-like. 2) Epigyne without median plate and concavity; Cos located medially, higher than the spermathecae; CD (the left one from the ventral view) straight or slightly clockwise from the CO; spermathecae close together or separated by less than half a spermatheca’s diameter.	1) *Otacilia bifurcata* Dankittipakul & Singtripop, 2014 2) *Otacilia christae* Jäger & Wunderlich, 2012 3) *Otacilia flexa* Fu, Zhang & Zhang, 2016* 4) *Otacilia longituba* Wang, Zhang & Zhang, 2012* 5) *Otacilia loriot* Jäger & Wunderlich, 2012 6) *Otacilia microstoma* Wang et al., 2015* 7) *Otacilia mingsheng* Yang, Wang & Yang, 2013* 8) *Otacilia mira* Fu, Zhang & Zhang, 2016* 9) *Otacilia mustela* Kamura, 2008 10) *Otacilia namkhan* Jäger & Wunderlich, 2012 11) *Otacilia parva* Deeleman-Reinhold, 2001 12) *Otacilia simianshan* Zhou, Wang & Zhang, 2013* 13) *Otacilia vangvieng* Jäger & Wunderlich, 2012 14) *Otacilia zebra* Deeleman-Reinhold, 2001 15) *Otacilia curvata* sp. n.* 16) *Otacilia submicrostoma* sp. n.*
*pseudostella*	1) Palpal organ without a distinct TA; an apophysis present near embolic base (PEA); embolus needle-like. 2) Epigyne without indistinct median plate, but with a pair of shallow concavities; Cos located anteriorly or medially, higher than the spermathecae; spermathecae separated by more than one spermatheca’s diameter.	1) *Otacilia acuta* Fu, Zhang & Zhang, 2016* 2) *Otacilia aurita* Fu, Zhang & Zhang, 2016* 3) *Otacilia digitata* Fu, Zhang & Zhang, 2016* 4) *Otacilia leibo* Fu, Zhang & Zhang, 2016* 5) *Otacilia ovata* Fu, Zhang & Zhang, 2016* 6) *Otacilia pseudostella* Fu, Jin & Zhang, 2014* 7) *Otacilia stella* Kamura, 2005 8) *Otacilia vulpes* (Kamura, 2001) 9) *Otacilia zhangi* Fu, Jin & Zhang, 2014*
the others		1) *Otacilia luzonica* (Simon, 1898) 2) *Otacilia papilla* Dankittipakul & Singtripop, 2014 3) *Otacilia paracymbium* Jager & Wunderlich, 2012*

#### Otacilia hippocampa sp. n.

Taxon classificationAnimaliaAraneaePhrurolithidae

http://zoobank.org/C336230A-1FD5-435C-BA9C-13657E682F6F

Figs 1 , 2 , 3

##### Type material.

Holotype ♂, China, *Hunan Province*: Dao County, Qingtang Town, Dajiangyuan Village, Mt. Jiucailing (25°27'37.678"N, 111°21'12.499"E), 448 m a.s.l., 29 September 2015, Chi Jin leg. Paratypes: 2♀2♂, same data as for holotype.

##### Etymology.

The species name is taken from the Latin generic name of the seahorse, “*Hippocampus*”, referring to the seahorse-shaped internal ducts (copulatory duct, connecting tube and spermatheca) in the female epigyne; adjective.

##### Diagnosis.

The male can be distinguished from all other *armatissima* group species, except *Otacilia bicolor* Jäger & Wunderlich, 2012, *Otacilia onoi* Deeleman-Reinhold, 2001 and *Otacilia truncata* Dankittipakul & Singtripop, 2014, by having a long DTA and can be distinguished from these three species by the absence of conductor (Figs 2A–D, 3A–C). The female of the new species can be easily distinguished from all of the other *armatissima* group species by the seahorse-shaped internal ducts (except the bursae), whereas they are S-shaped or crescent-shaped in the other congeners (Figs 2E–G, 3D–E).

##### Description.

**Figure 1.**
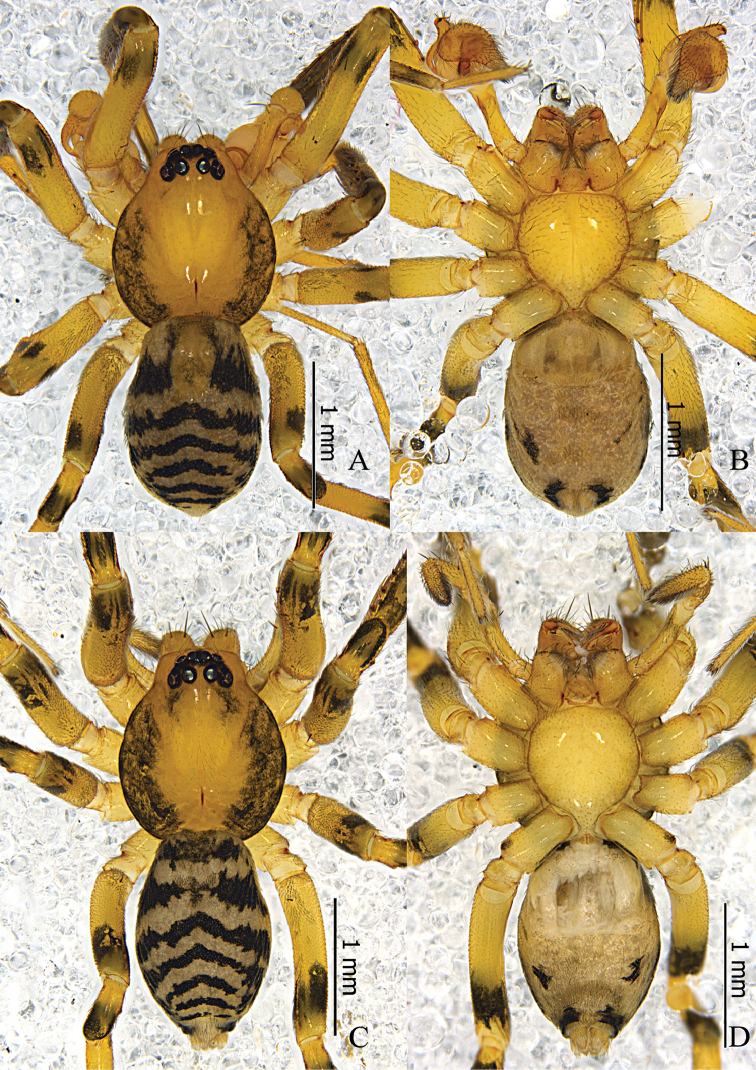
*Otacilia hippocampa* sp. n. **A** male habitus, dorsal view **B** same, ventral view **C** Female habitus, dorsal view **D** same, ventral view.

Male (Fig. 1A–B). Total length 2.17–2.65 (n = 3). Holotype: body 2.65 long; carapace 1.28 long, 1.12 wide; abdomen 1.37 long, 0.90 wide. Carapace yellowish brown, with black marginal bands; fovea longitudinal, brown. Eye diameters: AME 0.08, ALE 0.09, PME 0.08, PLE 0.09. Eye interdistances: AME–AME 0.02, AME–ALE 0.01, PME–PME 0.09, PME–PLE 0.05, ALE–PLE 0.07. MOA 0.19 long, front 0.17 wide, back 0.25 wide. Clypeus 0.14 high. Chelicerae with two strong anterior bristles; promargin with three well-separated teeth, and retromargin with two teeth close to each other. Labium and sternum dark yellow. Legs light yellowish brown; all femora with distal black distal annulus; patellae I–II black, patellae III–IV with distal black annulus; tibiae I–II almost all black, tibiae III–IV with black distal annulus; metatarsi I–II distal half part black, metatarsi III–IV with black distal annulus. Measurements of legs: leg I 4.95 (1.29, 0.49, 1.39, 1.23, 0.55), II 4.09 (1.11, 0.46, 1.01, 0.98, 0.53), III 3.59 (0.91, 0.44, 0.74, 0.94, 0.56), IV 5.19 (1.36, 0.45, 1.14, 1.49, 0.75). Leg formula: 4123. Femur I with two dorsal spines and three prolateral spines, femur II with one dorsal spine and two prolateral spines, femur III lacks dorsal spine, femur IV with one dorsal spine; tibia I with six proventral spines and seven retroventral spines, tibia II with six pairs of ventral spines; metatarsus I with four pairs of ventral spines, metatarsus II with four proventral spines and three retroventral spines. Femora I–III lack dorsal spines, femur IV four with one dorsal spine, femur I with three prolateral spines; tibia I with six proventral spines and seven retroventral spines, tibia II with six proventral spines and five retroventral spines; metatarsus I with four pairs of ventral spines, metatarsus II with four proventral spines and three retroventral spines. Abdomen oval, dorsum light grey, with several chevron-like black stripes, anterior half with a small dorsal scutum; venter light grey.

**Figure 2.**
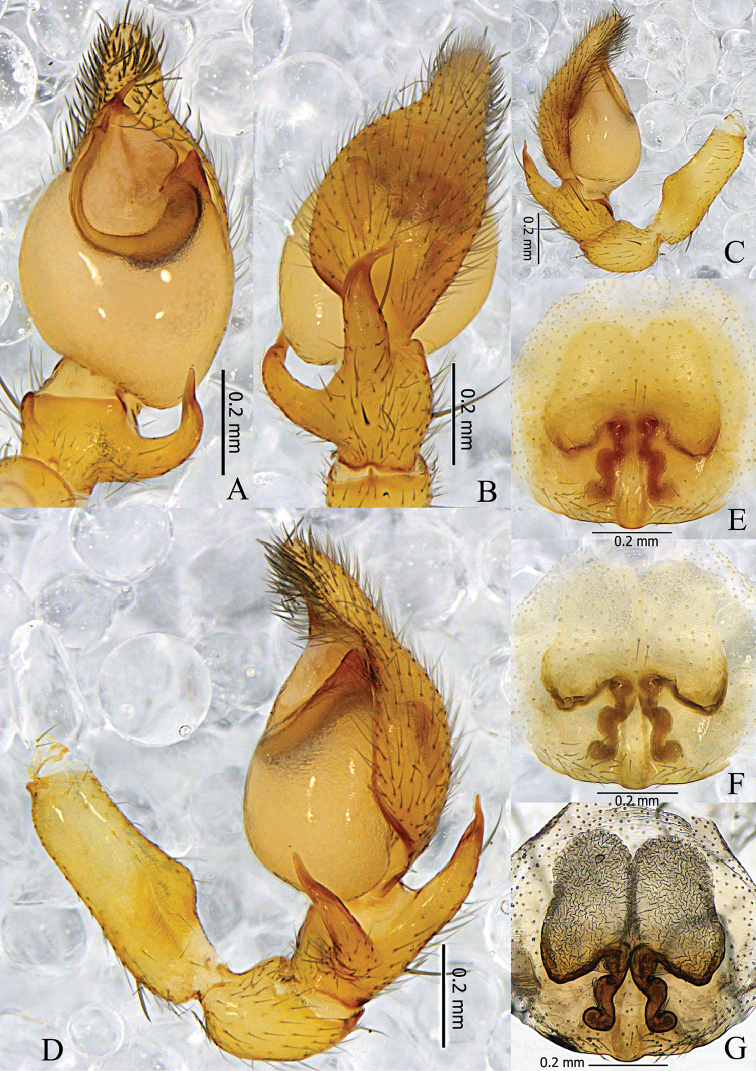
*Otacilia hippocampa* sp. n. **A** left male palp, ventral view **B** same, dorsal view **C** same, prolateral view **D** same, retrolateral view **E** epigyne, ventral view **F** same, cleared by potassium hydroxide, ventral view **G** vulva, dorsal view.

**Figure 3.**
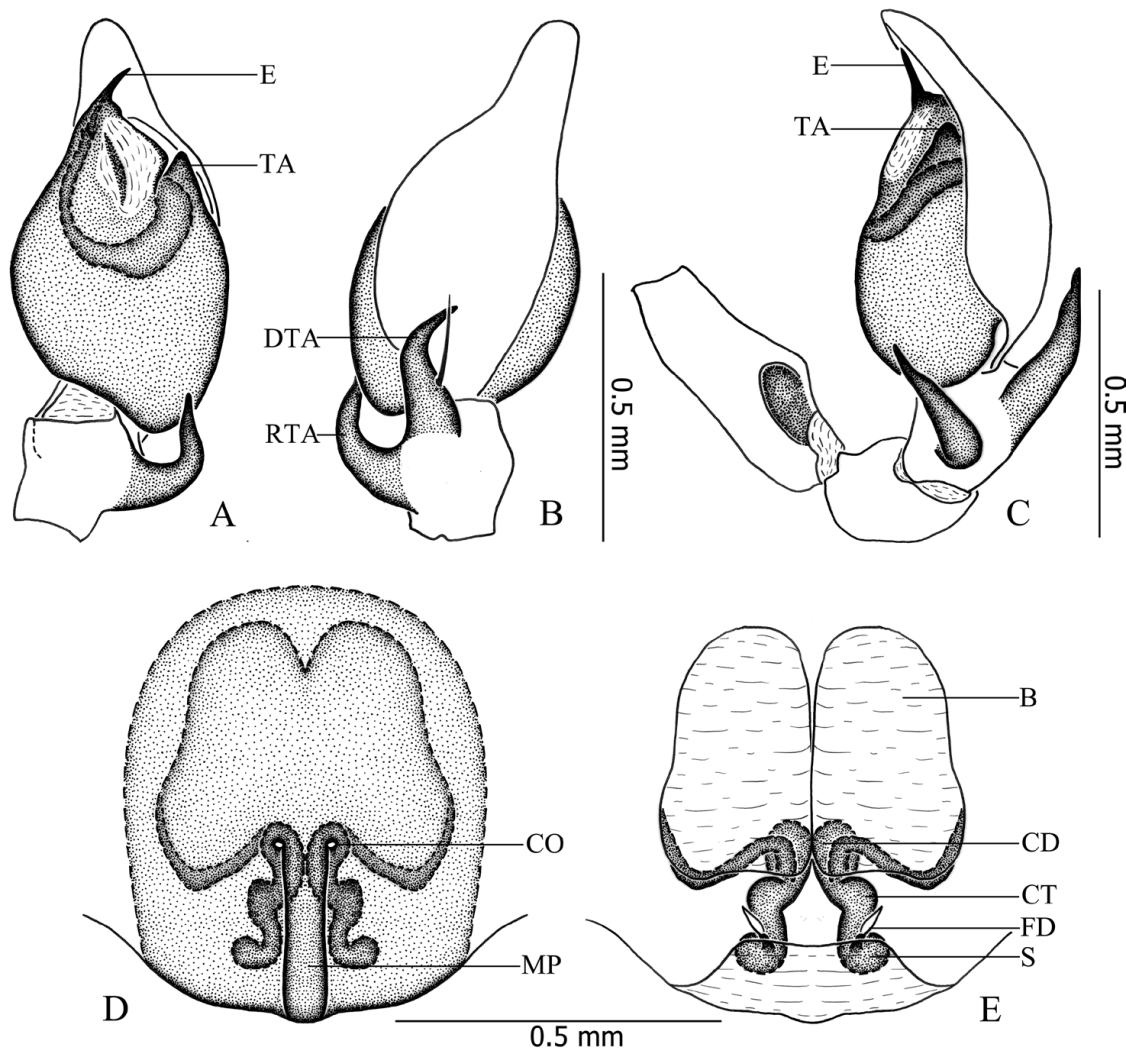
*Otacilia hippocampa* sp. n. **A** left male palp, ventral view **B** same, dorsal view **C** same, retrolateral view **D** epigyne, ventral view **E** vulva, dorsal view. Scale bars equal for **A** and **B**, equal for **D** and **E**.

Palp (Figs 2A–D, 3A–C). Femur distally with an inflated hump on ventral side and a retrolateral concavity. RTA basally thick, tapering to a sharp apex, bent prolaterally. DTA shaped similarly to RTA, with one spine basally. Embolus short, needle-like. Conductor absent. Tegular apophysis triangular, sclerotized.

Female (Fig. 1C–D). Total length 2.56–2.96 (n = 2). One paratype: body 2.96 long; carapace 1.47 long, 1.22 wide; abdomen 1.49 long, 0.99 wide. Eye diameters: AME 0.07, ALE 0.09, PME 0.08, PLE 0.10. Eye interdistances: AME–AME 0.03, AME–ALE 0.01, PME–PME 0.09, PME–PLE 0.06, ALE–PLE 0.09. MOA 0.22 long, front 0.18 wide, back 0.27 wide. Clypeus 0.13 high. Leg measurements: I 5.40 (1.41, 0.58, 1.54, 1.32, 0.55); II 4.53 (1.21, 0.53, 1.16, 1.04, 0.59); III 3.96 (1.04, 0.46, 0.81, 1.02, 0.63); IV 5.63 (1.46, 0.50, 1.27, 1.60, 0.80). Leg formula: 4123. Leg spination as in male. Abdomen light grey, anterior half lacks dorsal scutum. Other characters as in male.

Epigyne (Figs 2E–F, 3D): median plate narrow, with parallel lateral margin; copulatory openings situated centrally, tiny and pore-like. Vulva (Figs 2G, 3E–F): copulatory ducts short, connected with a pair of slender tubes leading to the large, transparent ovoid bursae; spermathecae located posteriorly, small and ovoid, separated by more than one spermatheca’s diameter; connecting tubes curved and sigmoid. Glandular appendages absent.

##### Distribution.

Known only from the type locality, Hunan, China (Fig. 13).

#### Otacilia yangmingensis sp. n.

Taxon classificationAnimaliaAraneaePhrurolithidae

http://zoobank.org/9FA1C1B9-0F0B-455C-B1D8-E17B2897AA68

Figs 4 , 5 , 6

##### Type material.

Holotype ♂, China, *Hunan Province*: Shuangpai County, Mt. Yangming, Wanshou Temple (26°06'27.490"N, 111°55'19.186"E), 1375 m a.s.l., 26 September 2015, Chi Jin leg. Paratypes: 5♀4♂, same data as for holotype; 1♂, Shuangpai County, Mt. Yangming, Hongjun Pavilion (26°04'34.924"N, 111°56'19.223"E), 1324 m a.s.l., 27 September 2015, Xiangbo Guo leg.; 1♂, Jiangyong County, Qianjiadong Town, Daboshui (25°24'25.70"N, 111°19'04.33"E), 224 m a.s.l., 3 October 2015, Jingchao He leg.

##### Etymology.

The species name refers to the holotype locality; adjective.

##### Diagnosis.

The male can be distinguished from all other *armatissima* group species, except *Otacilia macrospora* Fu. Zhang & Zhang, 2016, by the RTA base with a triangular process dorsally and by the absent DTA and can be distinguished from it by the long needle-like embolus (embolus stout and hook-shaped in *Otacilia macrospora*) and the thumb-shaped tegular apophysis (tegular apophysis sickle-shaped in *Otacilia macrospora*) (Figs 5A–B, 6A–B; Fu et al. 2016a: figs 16, 18, 22–23). The female of the new species can be distinguished from all other *armatissima* group species, except *Otacilia macrospora* Fu, Zhang & Zhang, 2016, by the copulatory openings connected with a pair of shallow concavities anteriorly, and the concavities have distinct anterior and inner lateral margins, and can be distinguished from it by the median plate (narrower than that of *Otacilia macrospora*) and bursae (long ovoid, whereas they are spherical in *Otacilia macrospora*) (Figs 5E–G, 6D–E; Fu et al. 2016a: figs 20–21, 25–26).

##### Description.

**Figure 4.**
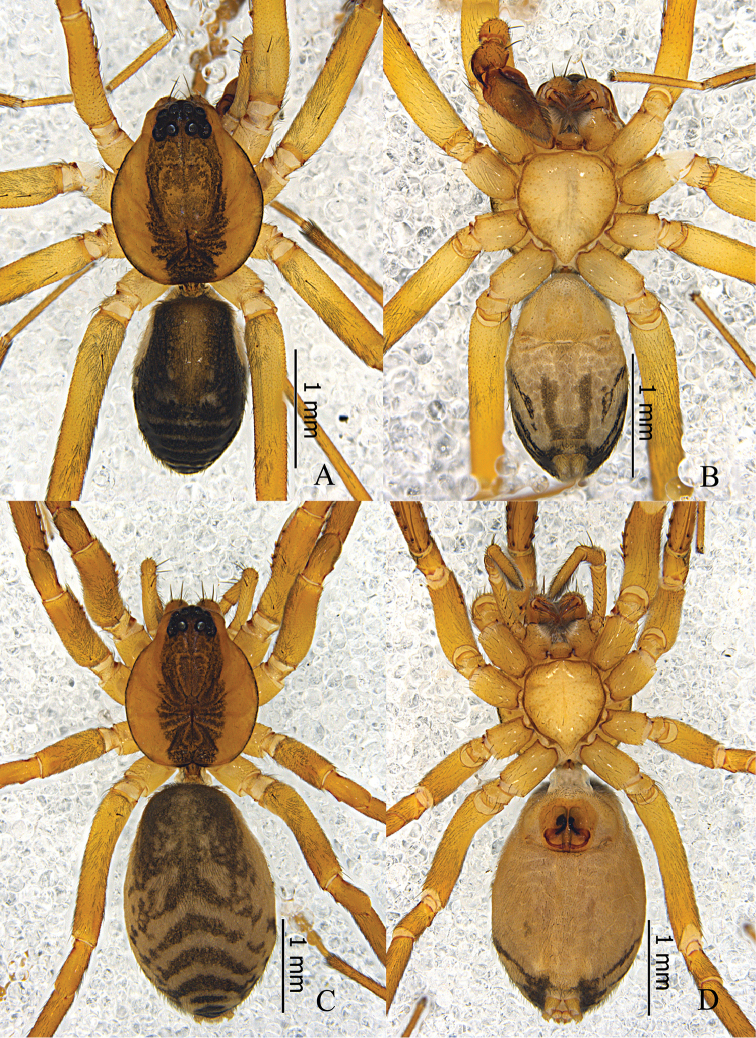
*Otacilia yangmingensis* sp. n. **A** male habitus, dorsal view **B** same, ventral view **C** Female habitus, dorsal view **D** same, ventral view.

Male (Fig. 4A–B). Total length 3.04–3.16 (n = 7). Holotype: body 3.16 long; carapace 1.58 long, 1.31 wide; abdomen 1.58 long, 0.97 wide. Carapace light yellowish brown, lateral margin black, middle with broad longitudinal black stripe, from ocular area to the posterior margin of carapace; fovea longitudinal, distinct. Eye diameters: AME 0.11, ALE 0.12, PME 0.10, PLE 0.10. Eye interdistances: AME–AME 0.03, AME–ALE 0.01, PME–PME 0.10, PME–PLE 0.05, ALE–PLE 0.09. MOA 0.25 long, front 0.23 wide, back 0.27 wide. Clypeus 0.14 high. Chelicerae with two strong anterior bristles; promargin with three well separated teeth and retromargin with six denticles close to each other. Labium and sternum light yellow. Legs light yellowish brown. Leg measurements: leg I 6.65 (1.66, 0.61, 1.89, 1.65, 0.84), II 5.36 (1.42, 0.47, 1.43, 1.29, 0.75), III 4.56 (1.22, 0.49, 1.01, 1.20, 0.64), IV 7.38 (2.00, 0.58, 1.75, 2.08, 0.97). Leg formula: 4123. Femur I with two dorsal spines and four prolateral spines, femur II with one dorsal spine and two prolateral spines, femora III–IV with one dorsal spine; tibia I with seven proventral spines and eight retroventral spines, tibia II with seven pairs of ventral spines; metatarsi I–II with four pairs of ventral spines. Abdomen oval, dorsum black, anterior half with a narrow dorsal scutum, posterior half with several black transversal stripes; venter light grey, with black longitudinal stripes.

**Figure 5.**
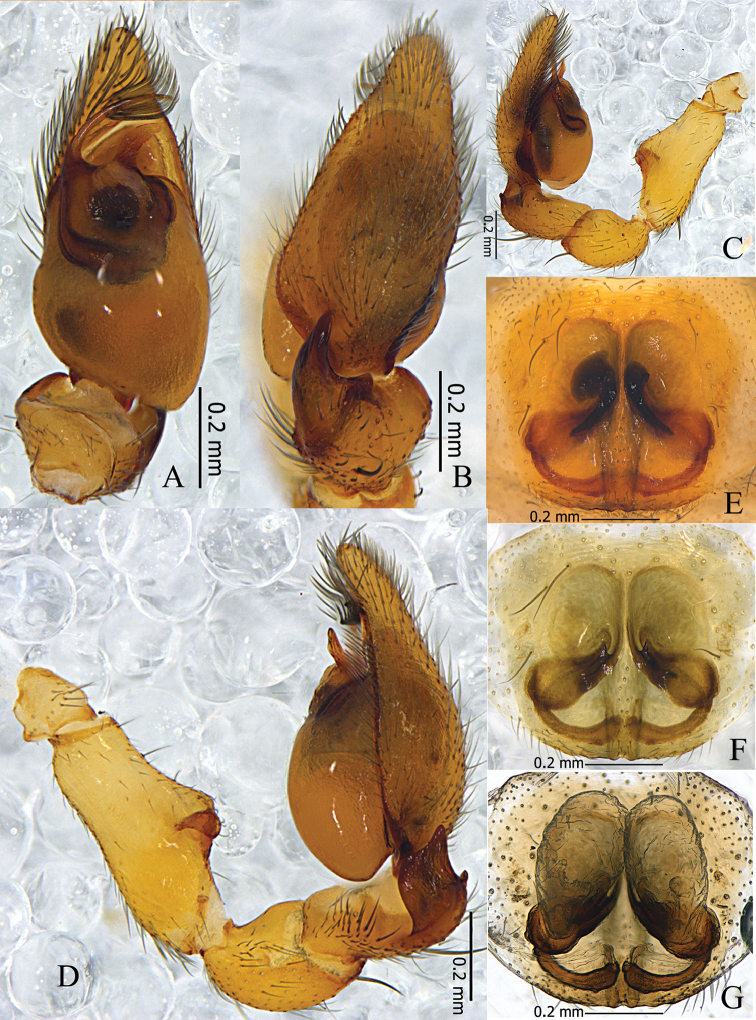
*Otacilia yangmingensis* sp. n. **A** left male palp, ventral view **B** same, dorsal view **C** same, prolateral view **D** same, retrolateral view **E** epigyne, ventral view **F** same, cleared by potassium hydroxide, ventral view **G** vulva, dorsal view.

**Figure 6.**
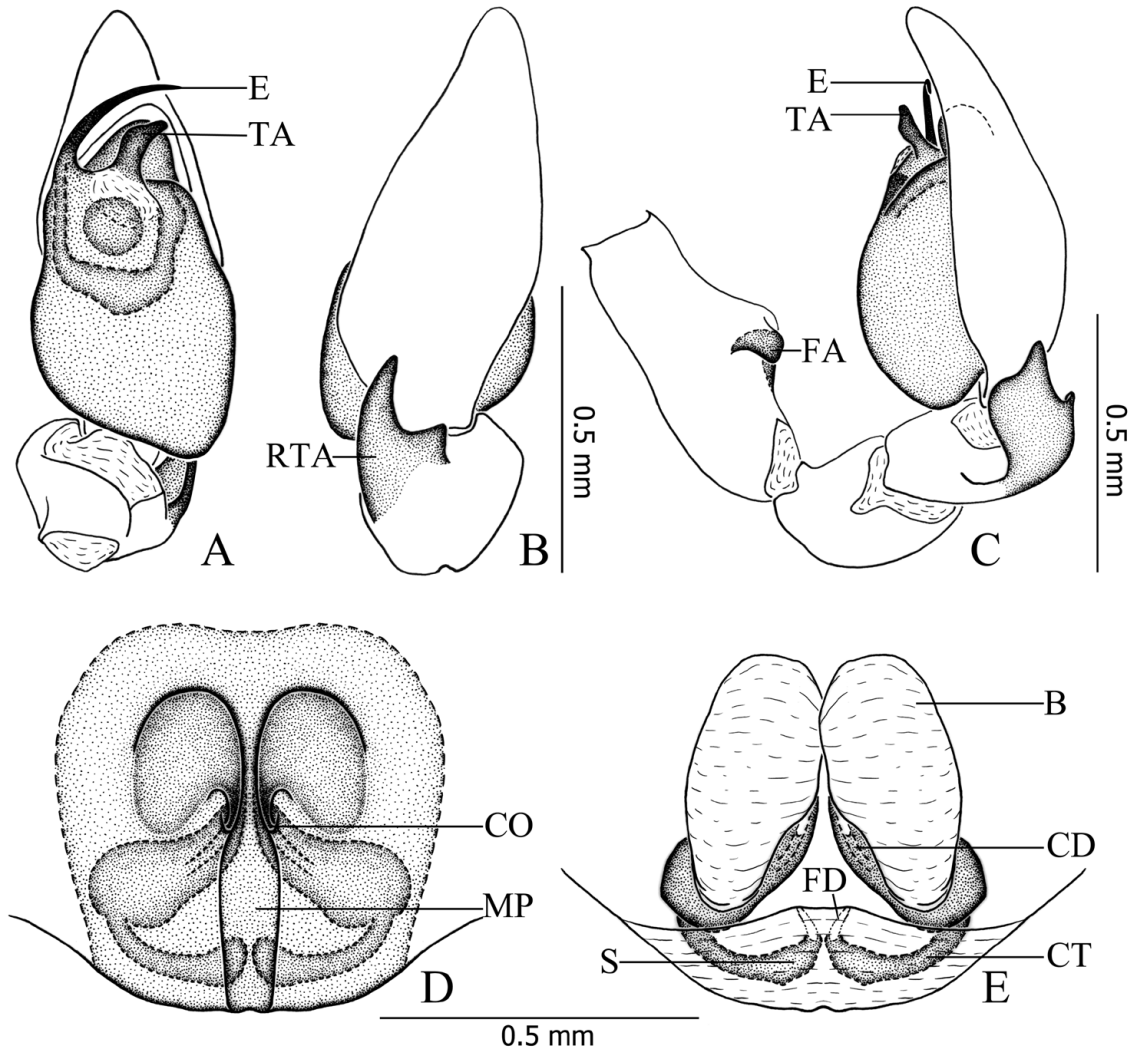
*Otacilia yangmingensis* sp. n. **A** left male palp, ventral view **B** same, dorsal view **C** same, retrolateral view **D** epigyne, ventral view **E** vulva, dorsal view. Scale bars equal for **A** and **B**, equal for **D** and **E**.

Palp (Figs 5A–D, 6A–C). Femur distally with an apophysis on ventral side anda retrolateral concavity. RTA broad, with sharp apex, base with a triangular process dorsally. Embolus slender, needle-like, slightly curved. Tegular apophysis sclerotized and thumb-shaped, situated at the apex of the bulb, separate from the embolus base.

Female (Fig. 4C–D). Total length 3.27–4.29 (n = 5). One paratype: body 4.29 long; carapace 1.72 long, 1.44 wide; abdomen 2.57 long, 1.62 wide. Carapace yellowish brown. Eye diameters: AME 0.11, ALE 0.10, PME 0.09, PLE 0.10. Eye interdistances: AME–AME 0.04, AME–ALE 0.01, PME–PME 0.11, PME–PLE 0.05, ALE–PLE 0.11. MOA 0.26 long, front 0.24 wide, back 0.31 wide. Clypeus 0.13 high. Leg measurements: I 6.63 (1.71, 0.65, 1.91, 1.56, 0.80); II 5.48 (1.44, 0.60, 1.42, 1.28, 0.74); III 4.67 (1.24, 0.56, 0.97, 1.20, 0.70); IV 7.29 (1.90, 0.63, 1.76, 2.00, 1.00). Leg formula: 4123. Femur I with two dorsal spines and four prolateral spines, femur II with one dorsal spine and three prolateral spines, femora III–IV with one dorsal spine; tibia I with eight pairs of ventral spines, tibia II with eight proventral spines and seven retroventral spines; metatarsus I with four pairs of ventral spines, metatarsus II with four proventral spines and three retroventral spines. Abdomen light grey, anterior half without dorsal scutum, posterior half dark with several indistinct chevron-like black stripes dorsally. Other characters as in male.

Epigyne (Figs 5E–F, 6D): median plate narrow, with parallel lateral margin; copulatory openings situated centrally, covered with mating plugs (Fig. 5E), connected with a pair of shallow concavities anteriorly, and the concavities have distinct anterior and inner lateral margins. Vulva (Figs 5G, 6E): copulatory ducts thick, posteriorly swollen, connected to a pair of large, transparent long ovoid bursae; spermathecae located posteriorly and small, close to each other; bursae and spermathecae connected by slender, slightly curved connecting tubes.

##### Distribution.

Known only from the type localities, Hunan, China (Fig. 13).

#### Otacilia curvata sp. n.

Taxon classificationAnimaliaAraneaePhrurolithidae

http://zoobank.org/EFCA1B66-8035-41A4-BB6F-634B4F16BB01

Figs 7 , 8 , 9

##### Type material.

Holotype ♂, China, *Hunan Province*: Shuangpai County, Mt. Yangming, around the Forest Park Service (26°03'36.698"N, 111°56'12.707"E), 539 m a.s.l., 24 September 2015, Chi Jin leg. Paratypes: 5♀3♂, same data as for holotype; 2♀4♂, Shuangpai County, Mt. Yangming, Wanshou Temple (26°06'27.490"N, 111°55'19.186"E), 1375 m a.s.l., 26 September 2015, Chi Jin leg.; 2♀1♂, Shuangpai County, Mt. Yangming, Hongjun Pavilion (26°04'34.924"N, 111°56'19.223"E), 1324 m a.s.l., 27 September 2015, Xiangbo Guo and Jingchao He leg.

##### Etymology.

The specific name is derived from the Latin “curvatus”, meaning curved and refers to the shape of the DTA of the male palp; adjective.

##### Diagnosis.

The male can be distinguished from all other *longituba* group species, except *Otacilia bifurcata* Dankittipakul & Singtripop, 2014, *Otacilia loriot* Jäger & Wunderlich, 2012 and *Otacilia namkhan* Jäger & Wunderlich, 2012, by having a long RTA and a long DTA and can be distinguished from them by the needle-like embolus (embolus of these three species claw-like, knife-shaped and semicircular respectively) (Figs 8A–D, 9A–C). The female of the new species can be easily distinguished from all of the other *longituba* group species, except *Otacilia microstoma* Wang et al., 2015, by the copulatory ducts longitudinal and close together, and it can be distinguished from *Otacilia microstoma* by the present of glandular appendages and sigmoid connecting tubes (glandular appendages absent and connecting tubes V-shaped in *Otacilia microstoma*) (Figs 8E–G, 9D–E; Wang et al. 2015: figs 1D–E, 2F–G).

##### Description.

**Figure 7.**
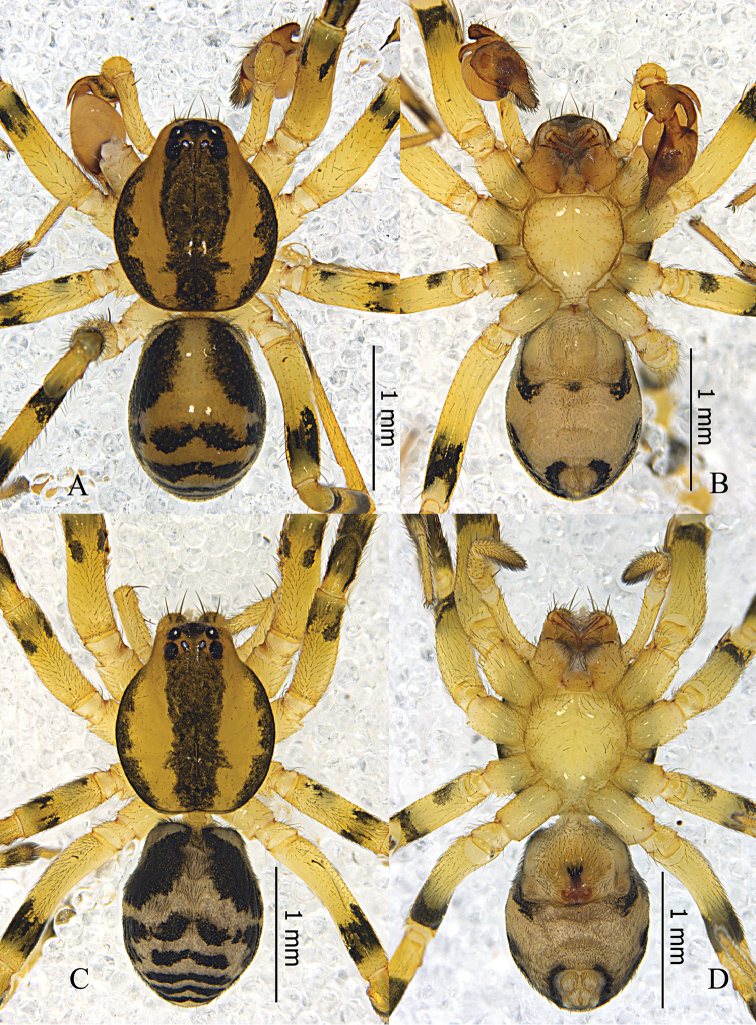
*Otacilia curvata* sp. n. **A** male habitus, dorsal view **B** same, ventral view **C** female habitus, dorsal view **D** same, ventral view.

Male (Fig. 7A–B). Total length 2.51–2.80 (n = 5). Holotype: body 2.67 long; carapace 1.37 long, 1.15 wide; abdomen 1.30 long, 0.96 wide. Carapace yellowish brown, with black marginal bands; middle with broad longitudinal black stripe, from ocular area to the posterior margin of carapace; fovea longitudinal, dark brown. Diameter of eyes: AME 0.08, ALE 0.09, PME 0.06, PLE 0.10. Eye interdistances: AME–AME 0.03, AME–ALE 0.01, PME–PME 0.08, PME–PLE 0.06, ALE–PLE 0.05. MOA 0.19 long, front 0.19 wide, back 0.20 wide. Clypeus 0.12 high. Chelicerae with two strong anterior bristles; promargin with three well-separated teeth and retromargin with five denticles close to each other. Labium and sternum dark yellow. Legs light yellowish brown; all femora with distal black annulus; patellae I–II all black, patellae III–IV absenting black patches; tibia I almost all black, tibiae II–IV with black proximal and distal annulus; metatarsus I distal half part black, metatarsi II–IV with black distal annulus. Measurements of legs: leg I 4.95 (1.31, 0.51, 1.42, 1.21, 0.50), II 3.90 (1.06, 0.40, 1.02, 0.91, 0.51), III 3.36 (0.86, 0.44, 0.68, 0.86, 0.52), IV 4.90 (1.29, 0.45, 1.09, 1.41, 0.66). Leg formula: 1423. Femora I–III lack dorsal spines, femur IV with one dorsal spine, femur I with three prolateral spines; tibia I with six proventral spines and seven retroventral spines, tibia II with six proventral spines and five retroventral spines; metatarsus I with four pairs of ventral spines, metatarsus II with four proventral spines and three retroventral spines. Abdomen oval, dorsum black, anterior half with a narrow dorsal scutum, posterior half with several chevron-like black stripes; venter light grey.

**Figure 8.**
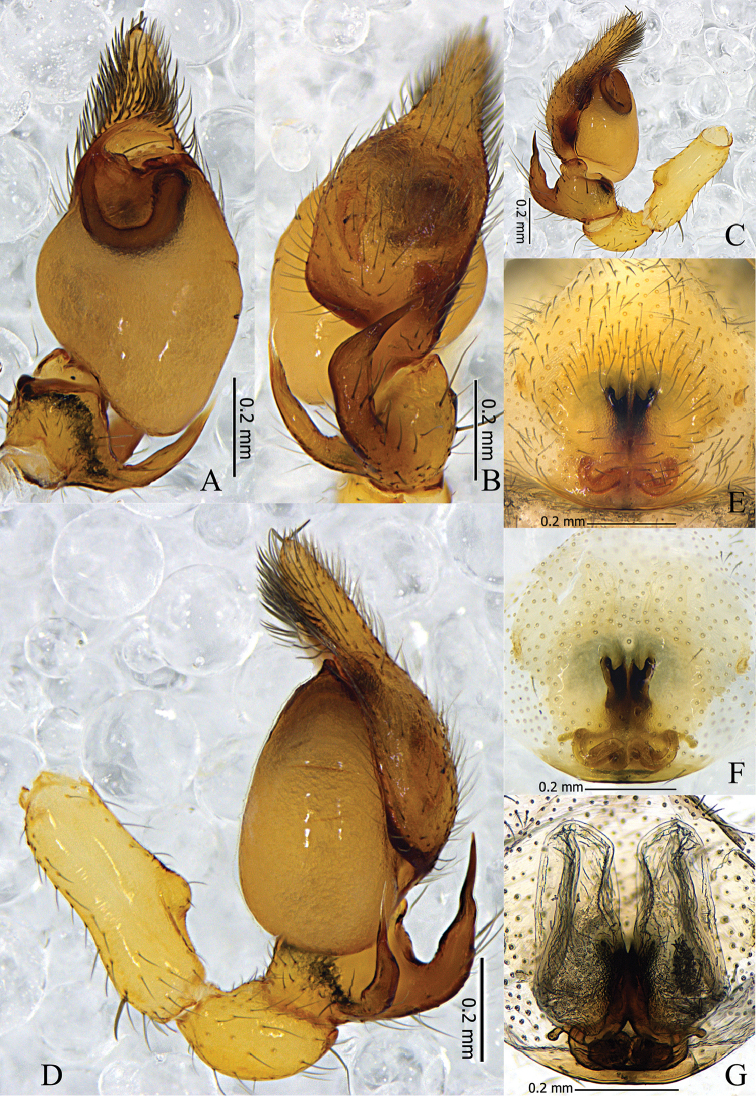
*Otacilia curvata* sp. n. **A** left male palp, ventral view **B** same, dorsal view **C** same, prolateral view **D** same, retrolateral view **E** epigyne, ventral view **F** same, cleared by potassium hydroxide, ventral view **G** vulva, dorsal view.

**Figure 9.**
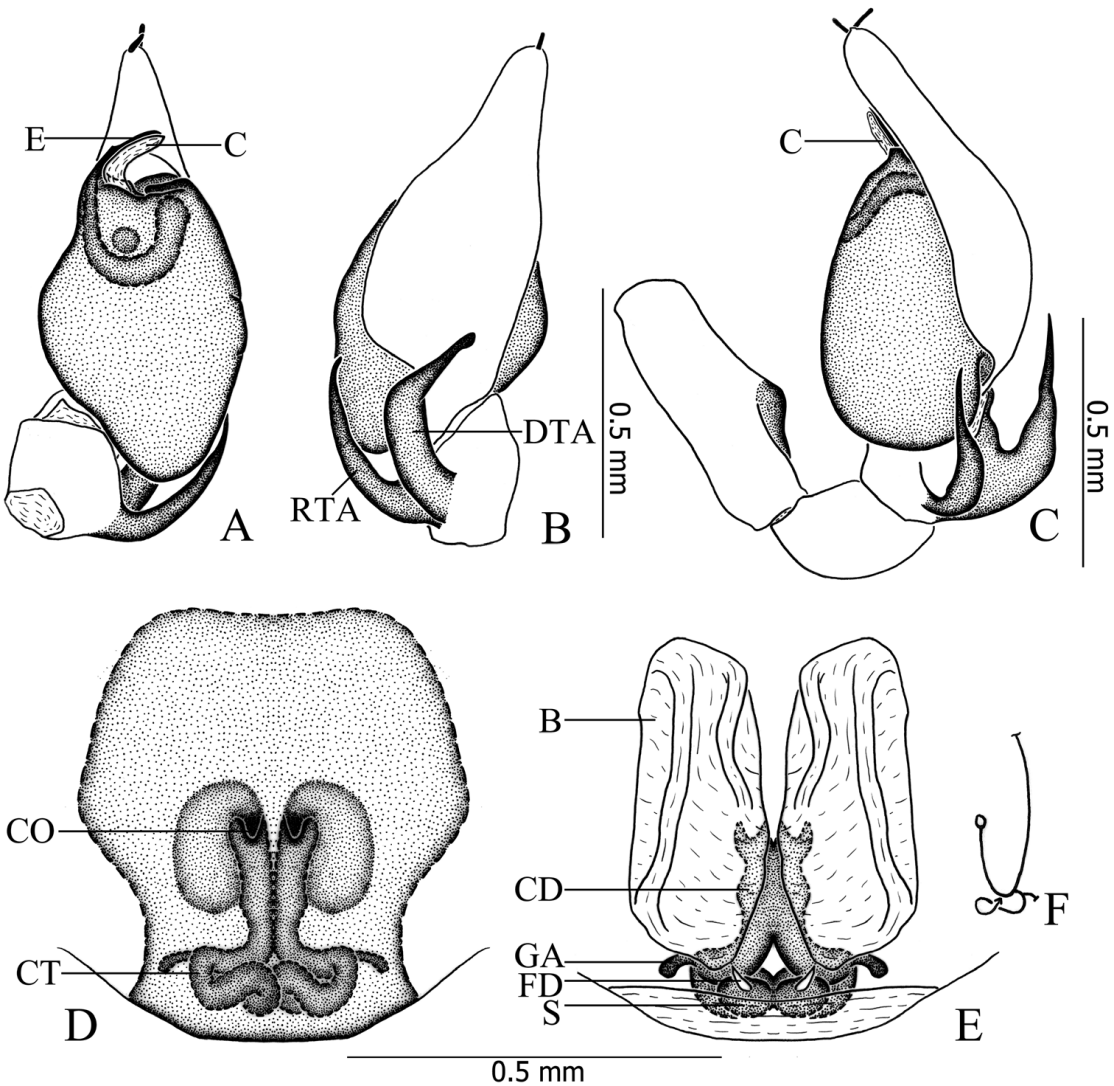
*Otacilia curvata* sp. n. **A** left male palp, ventral view **B** same, dorsal view **C** same, retrolateral view **D** epigyne, ventral view **E** vulva, dorsal view **F** schematic course of internal duct system. Scale bars equal for **A** and **B**, equal for **D** and **E**.

Palp (Figs 8A–D, 9A–C). Femur distally with an inflated hump on ventral side. RTA thick in proximal part and abruptly tapering at half of its length. DTA with anterior and posterior margins parallel in proximal part from the dorsal view, then abruptly curved to the prolateral side of bulb, tapering and with an enlarged blunt apex. Embolus short, needle-like. Conductor membranous, close to and as long as the embolus. Tegular apophysis absent but with a tegular ridge.

Female (Fig. 7C–D). Total length 2.77–2.85 (n = 7). One paratype: body 2.77 long; carapace 1.41 long, 1.20 wide; abdomen 1.36 long, 1.01 wide. Eye diameters: AME 0.08, ALE 0.09, PME 0.07, PLE 0.10. Eye interdistances: AME–AME 0.04, AME–ALE 0.01, PME–PME 0.07, PME–PLE 0.07, ALE–PLE 0.07. MOA 0.21 long, front 0.18 wide, back 0.19 wide. Clypeus 0.11 high. Leg measurements: I 5.11 (1.31, 0.54, 1.46, 1.29, 0.51); II 4.10 (1.10, 0.49, 1.03, 0.97, 0.51); III 3.46 (0.91, 0.43, 0.73, 0.86, 0.53); IV 5.08 (1.35, 0.50, 1.12, 1.41, 0.70). Leg formula: 1423. Femur II with one dorsal spines and two prolateral spines, tarsus II with six proventral spines and five retroventral spines, other segments with the same spination as male. Abdomen light grey, anterior half lacks dorsal scutum. Other characters as in male.

Epigyne (Figs 8E–F, 9D): median plate absent; copulatory openings situated centrally, tiny. and trumpet-shaped. Vulva (Figs 8G, 9E–F): copulatory ducts longitudinal, connecting with a pair of large, transparent, long, ovoid bursae; spermathecae located posteriorly, small and ovoid, close to each other; bursae and spermathecae connected by strong, curved, sigmoid connecting tubes. Glandular appendages present, as long as the diameter of one spermatheca.

##### Distribution.

Known only from the type localities, Hunan, China (Fig. 13).

#### Otacilia submicrostoma sp. n.

Taxon classificationAnimaliaAraneaePhrurolithidae

http://zoobank.org/64BE3E6B-B7E9-40EB-A1F0-39A01D85D844

Figs 10 , 11 , 12

##### Type material.

Holotype ♂, China, *Hunan Province*: Sangzhi County, Bamaoxi Town, Mt. Tianping, Watch Tower (29°47'11.854"N, 110°05'28.838"E), 1626 m a.s.l., 15 September 2015, Chi Jin leg. Paratypes: 11♀7♂, same data as for holotype; 19♀19♂, Sangzhi County, Bamaoxi Town, Mt. Tianping (29°46'07.921"N, 110°04'22.159"E), 1330 m a.s.l., 16 September 2015, Xiangbo Guo and Jingchao He leg.; 2♀6♂, Sangzhi County, Bamaoxi Town, Mt. Tianping (29°46'35.332"N, 110°05'54.474"E), 1520 m a.s.l., 17 September 2015, Chi Jin leg.

##### Etymology.

The species is named for its similarity to *Otacilia microstoma* Wang et al., 2015; adjectival.

##### Diagnosis.

The male can be distinguished from all other *longituba* group species, except *Otacilia mira* Fu, Zhang & Zhang, 2016, *Otacilia mustela* Kamura, 2008 and *Otacilia parva* Deeleman-Reinhold, 2001, by having only one tibial apophysis and no conductor and can be distinguished from them by the RTA base with a small triangular process (Figs 11A–D, 12A–C). The female of the new species can be easily distinguished from all of the other *longituba* group species by the long, S-shaped connecting peculiar tubes (Figs 11E–F, 12D).

##### Description.

**Figure 10.**
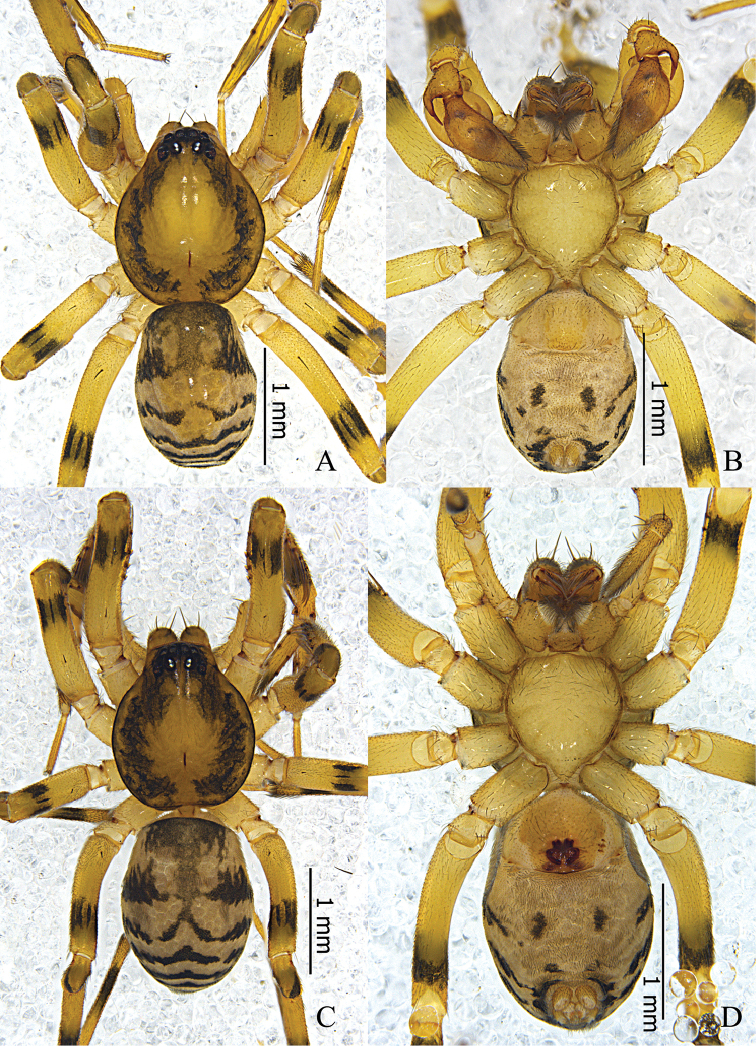
*Otacilia submicrostoma* sp. n. **A** male habitus, dorsal view **B** same, ventral view **C** female habitus, dorsal view **D** same, ventral view.

Male (Fig. 10A–B). Total length 2.65–2.99 (n = 33). Holotype: body 2.99 long; carapace 1.52 long, 1.29 wide; abdomen 1.47 long, 1.04 wide. Carapace yellowish brown, with black marginal bands; fovea longitudinal, brown. Eye diameters: AME 0.09, ALE 0.10, PME 0.09, PLE 0.10. Eye interdistances: AME–AME 0.04, AME–ALE 0.01, PME–PME 0.11, PME–PLE 0.05, ALE–PLE 0.08. MOA 0.22 long, front 0.21 wide, back 0.30 wide. Clypeus 0.15 high. Chelicerae with two strong anterior bristles; promargin with three well-separated teeth and retromargin with seven denticles close to each other. Labium and sternum dark yellow. Legs light yellowish brown, all femora, patellae, tibiae, metatarsi distally with black annulus. Measurements of legs: leg I 5.45 (1.42, 0.54, 1.55, 1.30, 0.64), II 4.54 (1.20, 0.47, 1.18, 1.08, 0.61), III 3.96 (1.04, 0.44, 0.83, 1.03, 0.62), IV 5.72 (1.56, 0.50, 1.26, 1.57, 0.83). Leg formula: 4123. Femur I with two dorsal spines and four prolateral spines, femur II with one dorsal spine and one prolateral spine, femora III–IV with one dorsal spine; tibia I with six proventral spines and seven retroventral spines, tibia II with six pairs of ventral spines; metatarsus I with four pairs of ventral spines, metatarsus II with four proventral spines and three retroventral spines. Abdomen oval, dorsum black, anterior half with a narrow dorsal scutum, posterior half with several black transverse stripes; venter light grey, with black scattered patches.

**Figure 11.**
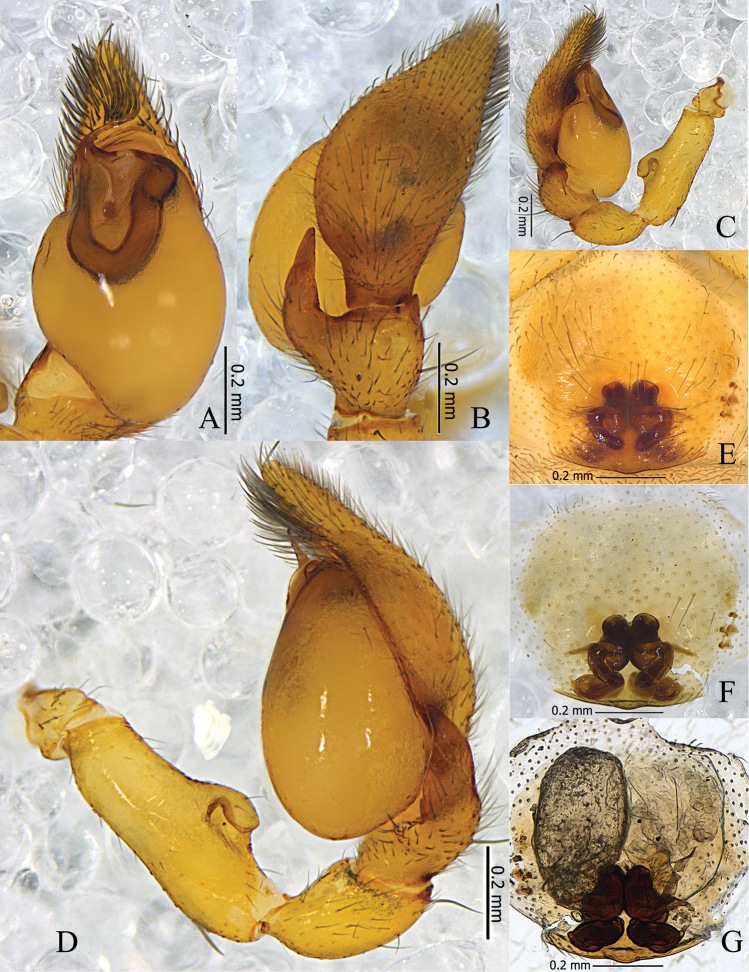
*Otacilia submicrostoma* sp. n. **A** left male palp, ventral view **B** same, dorsal view **C** same, prolateral view **D** same, retrolateral view **E** epigyne, ventral view **F** same, cleared by potassium hydroxide, ventral view **G** vulva, dorsal view.

**Figure 12.**
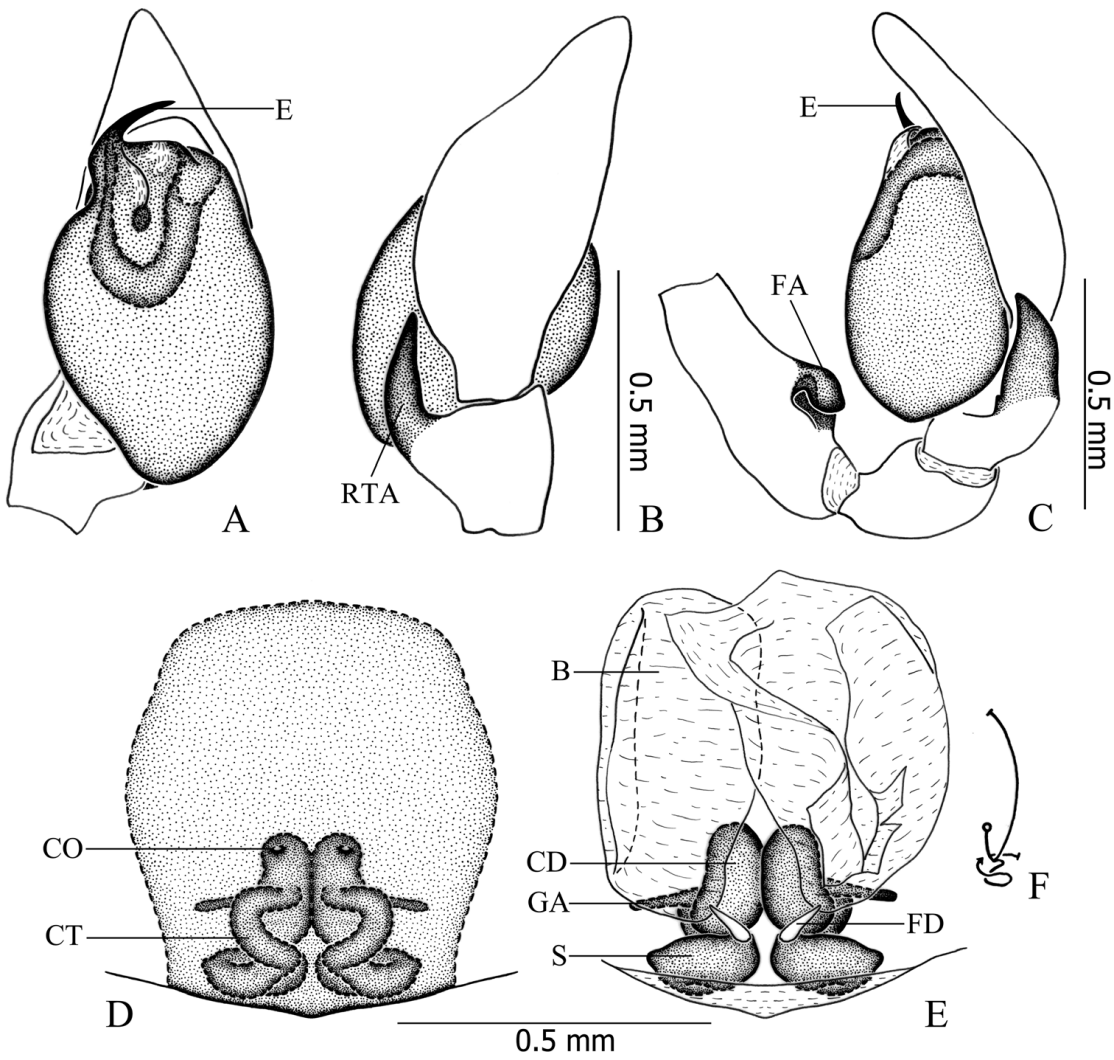
*Otacilia submicrostoma* sp. n. **A** left male palp, ventral view **B** same, dorsal view **C** same, retrolateral view **D** epigyne, ventral view **E** vulva, dorsal view **F** schematic course of internal duct system. Scale bars equal for **A** and **B**, equal for **D** and **E**.

Palp (Figs 11A–D, 12A–C). Femur distally with an apophysis on ventral side anda retrolateral concavity. RTA broad, with relatively sharp apex extending along the cymbium retrolaterally, basally with a small triangular process. Embolus slender, needle-like. Tegular apophysis and conductor absent.

Female (Fig. 10C–D). Total length 3.02–3.48 (n = 32). One paratype: body 3.48 long; carapace 1.60 long, 1.36 wide; abdomen 1.88 long, 1.25 wide. Carapace yellowish brown. Eye diameters: AME 0.09, ALE 0.09, PME 0.10, PLE 0.10. Eye interdistances: AME–AME 0.04, AME–ALE 0.01, PME–PME 0.10, PME–PLE 0.06, ALE–PLE 0.09. MOA 0.25 long, front 0.20 wide, back 0.29 wide. Clypeus 0.14 high. Leg measurements: I 5.71 (1.47, 0.59, 1.64, 1.40, 0.61); II 4.87 (1.28, 0.52, 1.20, 1.23, 0.64); III 4.11 (1.09, 0.47, 0.85, 1.04, 0.66); IV 5.98 (1.60, 0.53, 1.33, 1.64, 0.88). Leg formula: 4123. Femur I with two dorsal spines and four prolateral spines, femur II with one dorsal spine and two prolateral spines, femora III–IV with one dorsal spine; tibiae and metatarsi I and II with the same spination as male. Abdomen light grey, anterior half lacking dorsal scutum, posterior half dark with several indistinct chevron-like black stripes dorsally. Other characters as in male.

Epigyne (Figs 11E–F, 12D): median plate absent; copulatory openings situated centrally, tiny and pore-like. Vulva (Figs 11G, 12E–F): copulatory ducts thick and short, connected with a pair of large, transparent, long, ovoid bursae; spermathecae located posteriorly, large and ovoid, close to each other; bursae and spermathecae connected by strongly curved, S-shaped connecting tubes. Glandular appendages present, as long as one spermatheca’s diameter.

##### Distribution.

**Figure 13.**
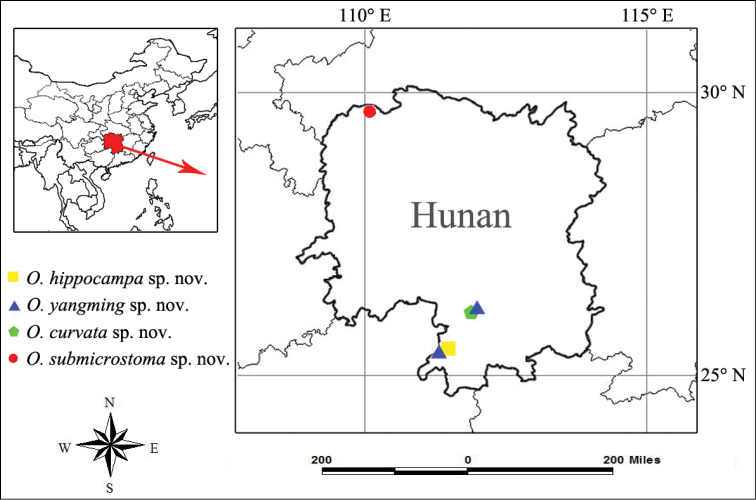
Distribution of new species the genus *Otacilia* from Hunan, China.

Known only from the type localities, Hunan, China (Fig. 13).

## Supplementary Material

XML Treatment for Otacilia

XML Treatment for Otacilia hippocampa

XML Treatment for Otacilia yangmingensis

XML Treatment for Otacilia curvata

XML Treatment for Otacilia submicrostoma
